# Influence and Longevity of a Microsurgery Course for Medical Students on Their Future Careers: A Retrospective Report of Up to 10 Years

**DOI:** 10.1055/a-2540-0917

**Published:** 2025-03-07

**Authors:** Lucas M. Ritschl, Alex Grabenhorst, Constantin Wolff, Katharina Pippich, Dorothea Dick, Pascal O. Berberat, Klaus-Dietrich Wolff, Andreas M. Fichter

**Affiliations:** 1 Department of Oral and Maxillofacial Surgery, Department of Clinical Medicine, TUM School of Medicine and Health, Technical University of Munich, Klinikum rechts der Isar, Munich, Germany; 2 Department of Clinical Medicine, TUM Medical Education Center, TUM School of Medicine and Health, Technical University of Munich, Munich, Germany

**Keywords:** microsurgery, microsurgical training, online survey, longevity, vascular anastomosis, chicken thigh

## Abstract

**Background:**

This study evaluates the influence and longevity of a microsurgery course on the future careers of medical students over a period of up to 10 years. The course, which has been well-established for over 15 years, aimed to impart fundamental microsurgical skills through practical exercises using nonbiological and biological models.

**Methods:**

This study was conducted as an anonymous online cross-sectional survey. Only students who have completed a microsurgical training course at our department within a 10 years period between 2013 and 2023 were eligible for this online survey. This survey aimed to analyze the subjective microsurgical skills at the time of the survey and the influence of the course on further career decisions and development.

**Results:**

A total of 300 former participants were eligible and 120 answered the survey. Key findings showed that 99.2% of participants rated the course content and the balance of practice to theory as appropriate, with 100% feeling confident in their microsurgical suturing abilities post-course. A significant 87.5% felt competent to perform vascular anastomoses, though only 63.9% felt confident about nerve coaptation. Statistical analysis indicated no significant gender differences in course evaluations, though some differences were noted between semester-accompanying and block course participants. In the survey, 54.2% of respondents reported using their microsurgical skills in their subsequent medical careers, and 50.4% indicated that the course influenced their medical specialization choices.

**Conclusion:**

The study concludes that early exposure to microsurgical training can significantly impact students' skills and career trajectories, supporting the integration of such courses into medical education curricula to enhance surgical training and professional development.

## Background

Reconstructive microsurgery is a critical discipline requiring skills ideally acquired through years of training on various nonliving and living models. 1 Free transplantation of complex tissue grafts and successful nerve coaptation are essential components of reconstructive surgery used in multiple surgical disciplines. The classical microsurgical curriculum includes a three-step approach with three levels of models: (1) nonbiological nonfunctional models (latex glove, silicon tube, synthetic vessel); (2) biological nonfunctional models (chicken thigh/wing); and (3) biological and functional models (rat). 2 3 4 5 However, not all interested surgeons have access to or the opportunity for microsurgery training during specialization or as specialists in departments/hospitals. 6 Additionally, workload, work time regulations, and work–life balance have increased the demand for effective training and assessment methods to optimize learning outside the clinical setting during residency and early specialization. 7 8 9 10 Consequently, various national and international microsurgery courses at high-volume centers involve course fees and costs for travel and accommodation. These courses involve practicing on different models, and the quality of the anastomoses can be assessed using several tests. 11 12 13 However, no animal is needed to learn skills such as correct needle guidance, careful tissue handling, and technically sound execution of a microsurgical suture. 7 Models like the latex glove, vessel loop, chicken thighs, or pig heart coronaries suffice for these initial steps in microsurgery and represent the large group of laboratory-based low-fidelity models. 7 14 Recently, there has been a trend away from biological and functional high-fidelity models toward sophisticated biological nonfunctional models like the perfused chicken thigh model, aligning with the 3Rs (replacement, reduction, and refinement).

A previous study demonstrated that the timing of exposure to microsurgery can influence success. Interestingly, Mücke et al found that young medical students sometimes performed better in standardized practical microsurgery tests than residents with several years of experience. 15 Various conclusions were drawn from this finding. First, it is advantageous to start young, as is the case in many practically demanding fields such as sports. Brain plasticity and the openness to learning complex and fine movements play significant roles. Additionally, it underscores the importance of early exposure to this subdiscipline for those interested in surgery.

In a further step, we aimed to critically evaluate our course in the context of overall medical training and the subjective perception of the microsurgical skills acquired. Additionally, we investigated whether former participants were able to utilize their microsurgical skills in their subsequent medical careers.

## Methods

### Student Microsurgical Course

The microsurgical training course over the analyzed 10-year period consisted of three types: (1) a regular semester-accompanying course (10–12 course days of 2 hours each); (2) a regular block course (5 consecutive days of 3 hours each); and (3) a course during the SARS-CoV-2 pandemic (online lecture, practical exercises at home with a smartphone and borrowed microsurgical instruments). One course day consists of an introductory lecture to impart knowledge 16 17 18 19 20 21 and set the course day's tasks. This was followed by live demonstrations of the exercises, and two course tutors then supervised six students for the practical part of the course. The course is offered as an elective during the clinical section between the third and fifth year of medical studies at the Technical University of Munich. Students can choose the course without restrictions regarding their personal expertise and experience in surgery.

**Fig. 1:**
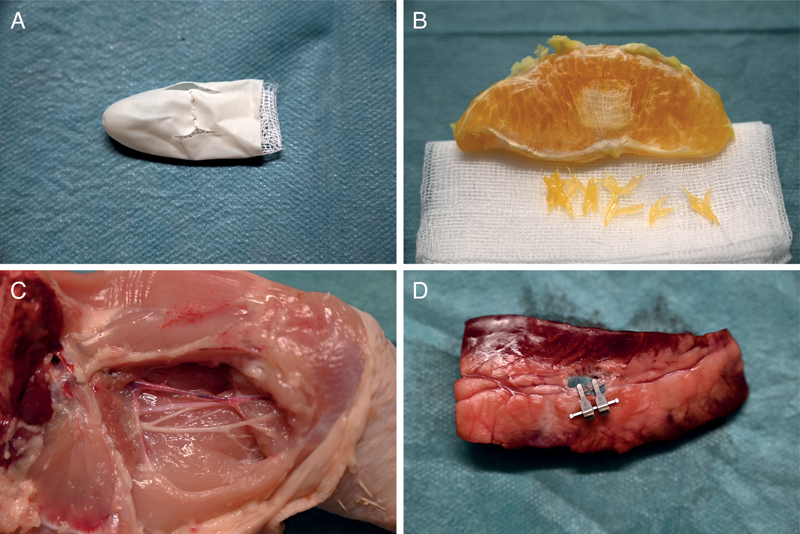
Exemplary illustration of the four laboratory-based low- and moderate-fidelity models used for microsurgical training: **(A)** latex glove, **(B)** orange model, **(C)** chicken model, and **(D)** pig heart coronaries.

The practical skills taught included atraumatic microsurgical tissue handling of vessels and nerve structures, 22 microsurgical suturing with different suture strengths ranging from 9-0 to 11-0 Ethilon (Ethilon®; Ethicon Division of Johnson & Johnson; Livingston, Scotland), end-to-end or end-to-side anastomosis, and nerve coaptation. Practical exercises involved tasks on established nonbiological and nonfunctional models such as latex gloves and oranges. 2 Later, biological and nonfunctional models, including chicken thighs and porcine heart coronaries, were used ( Fig. 1 ). Students could change the models individually at the end of the course to experience variation and individual learning control of the acquired manual skills. In this microsurgery course, no free flaps were lifted from the model, nor were any anatomies performed on living animals or in the cadaver.

### Evaluation

**Fig. 2:**
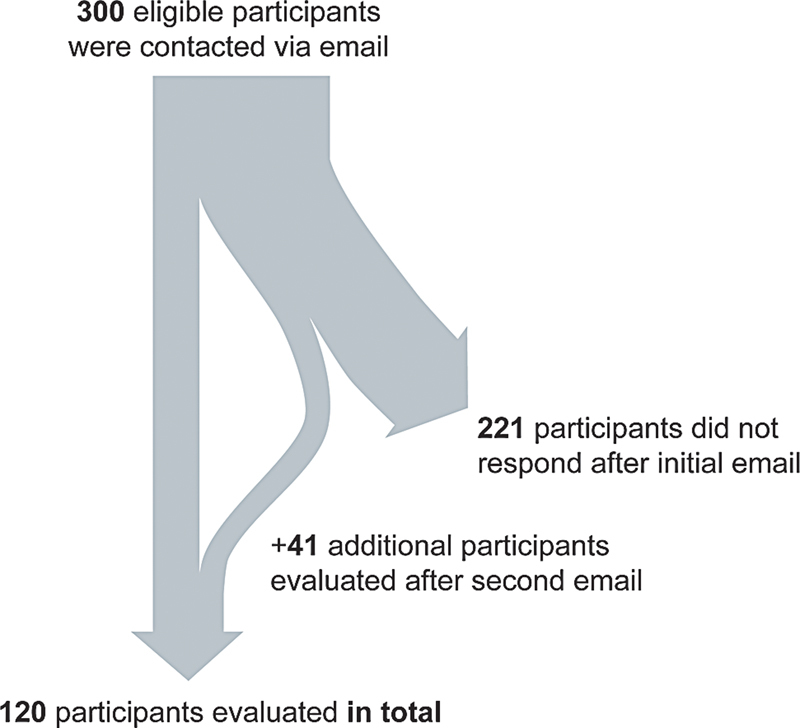
Flowchart of enrolled and analyzed participants.

Only students who have completed our well-established microsurgical training course 15 within a 10-year period between 2013 and 2023 were eligible for this online survey. This period was chosen because it ensured that the course instructor (first author) was constant. The former participants were contacted via email in two rounds using the web-based survey software evasys ( Fig. 2 ). An individual transaction authentication number (TAN) process ensured that each participant could only answer the survey once. The evaluation was completely anonymous; the completed forms could not be traced, and data were transmitted in encrypted form. Once an evaluation was submitted, it could not be accessed or edited again. If the evaluation was not submitted, the TAN remained active until the end of the evaluation period.

This study was conducted as an online cross-sectional survey using the web-based survey software evasys (evasys GmbH; 21337 Lüneburg, Germany). The software is ISO-27001 certified and thus guarantees a high level of quality management and information security. It also offers professionally managed hosting on secure, ISO-27001/27018-certified servers in Germany and is therefore GDPR-compliant.

### Statistical Analysis

All statistical tests were performed at an exploratory two-sided 5% significance level. No adjustments were made for multiple testing. The analysis was conducted using IBM SPSS 24 for Mac software (IBM Corp, Armonk, New York, United States). The Mann–Whitney U test was used to analyze the influence of gender or course type on the survey results.

## Results

### General Results

A total of 120 out of 300 eligible and contacted students responded to this online survey (40%), with a gender distribution of 65.8% female and 34.2% male participants. The median age of the participants was 23 years (ranging from 20 to 32) at the time of their course. The median age at the time of this online survey was 27 years (ranging from 23 to 38). Of the participants, 73.3% attended the semester-accompanying course, and 75.8% attended during the regular semester before and after the SARS-CoV-2 pandemic. Most of the students were in their third to fifth clinical semesters when they attended the course. The detailed distribution was as follows: 1.7% in the first clinical semester, 9.2% in the second clinical semester, 25.0% in the third clinical semester, 30.0% in the fourth clinical semester, 20.8% in the fifth clinical semester, and 12.5% in the sixth clinical semester. The most common way participants became aware of the course was through positive word of mouth from friends (42.5%), followed by mediTUM search (31.7%; mediTUM = the website with student organizing structure of the medical studies at the TUM School of Medicine and Health), other elective courses from our department (19.2%), student council (3.3%), and other sources also at 3.3%.

### Course-Specific Results

Of the participants, 99.2% rated the course content as appropriate. Similarly, 99.2% found the ratio of practice to theory balanced, and all felt that a sufficient amount of theory was taught. This resulted in a “very good” (equivalent to German school grade 1) evaluation from 97.5% and a “good” (equivalent to German school grade 2) evaluation from 2.5% of the participants.

Additionally, 100% felt confident in their ability to perform microsurgical suturing independently after the course, and 87.5% rated their acquired skills as sufficient to perform a patent microsurgical vascular anastomosis. In contrast, only 63.9% believed they could successfully perform nerve coaptation. The chicken thigh model was chosen as the favorite by 81.7%, followed by porcine heart coronary (15.0%), orange (2.5%), and latex glove (0.8%). However, pork heart coronaries were rated the most difficult/challenging by 41.2%, followed by the orange model (29.4%), chicken thighs (26.9%), and latex gloves (2.5%). A comparison of answers based on gender or course type is shown in Table 1 .

**Table 1:** Overview of the survey results in relation to gender (female *n* = 79 versus male = 41) or course type (semester-accompanying *n* = 88 versus block course *n* = 32)

Question	Gender, Yes/No (%)		*p* -value*	Course type, Yes/No (%)		*p* -value*
	Female	Male		Semester-accompanying	Block course	
Appropriate course content?	100.0/0.0	97.6/2.4	0.165	98.9/1.1	100.0/0.0	0.546
Appropriate ratio of practice/theory?	98.7/1.3	97.6/2.4	0.477	98.9/1.1	100.0/0.0	0.544
Sufficient theoretical knowledge imparted?	100.0/0.0	100.0/0.0	1.000	100.0/0.0	100.0/0.0	1.000
Felt safe performing a microsurgical suture?	100.0/0.0	100.0/0.0	1.000	100.0/0.0	100.0/0.0	1.000
Felt safe performing a microsurgical anastomosis?	88.6/11.4	85.4/14.6	0.612	86.4/13.6	90.6/9.4	0.534
Felt safe performing a nerve coaptation?	60.8/39.2	70.7/29.3	0.260	65.9/34.1	59.4/40.6	0.538
Further use of acquired manual skills?	58.2/41.8	46.3/53.7	0.217	54.5/45.5	53.1/46.9	0.891
Did you complete another elective course after the microsurgery course?	40.5/59.5	48.8/51.2	0.388	48.9/51.1	28.1/71.9	0.043
Did the course motivate you to visit OMFS?	57.0/43.0	65.9/34.1	0.348	61.4/38.6	56.3/43.7	0.615
Did the course motivate you to accomplish elective subject of the practical year at OMFS?	32.9/67.1	46.3/53.7	0.151	42.0/58.0	25.0/75.0	0.089
Had the course an influence on skills at the start of the career?	89.8/10.1	85.4/14.6	0.311	90.9/9.1	81.3/18.7	0.103

Abbreviation: OMFS, oral maxillofacial surgery.

Note: *Mann-Whitney U test with an exploratory two-sided 5% significance level.

### Results in Relation to the Further Development of Studies and Future Medical Focus or Career

Surgical experience prior to attending the course was evident in 80% of the participants, and 54.2% stated that they used the skills acquired after the course in activities such as a doctoral thesis or clinical work. After the microsurgery course, 43.3% completed a further course at our department, and 60.0% were motivated to do an internship in oral and maxillofacial surgery (OMFS), while 37.5% even completed their elective subject of their final practical year in our department. In total, 50.4% stated that the microsurgery course influenced their further medical focus or career (18.5% reported no influence, 31.1% were uncertain), and generally, 75.8% were motivated to pursue surgical work in the future. The course had a particular impact on skills at the start of their professional activities (e.g., safe handling of tissue, safe suturing) for 88.1% of the participants. The three most important factors influencing the choice of specialization were the final practical year, internships, and elective courses during studies.

A comparison of answers based upon gender or course type is shown in Table 1 .

## Discussion

The optimal design and implementation of a sophisticated surgical training program as part of medical education and specialization face many hurdles. Surgical instruments, time, specialist staff, and enthusiasm are needed to convince the new generation of doctors of the surgical possibilities. Establishing sound microsurgical training is even more challenging. Due to the high number of students per semester, such specialized training is difficult or even impossible to implement in compulsory courses as part of the standard curriculum because of equipment and personnel costs. Therefore, it is gratifying that our department has offered an elective subject for student training in microsurgery for over 15 years. The course graduates had different levels of training in terms of the number of semesters completed and the surgical skills acquired during their studies or clinical traineeships. However, majority of the participants were in the third to fifth clinical semesters and 80% stated that they had gained some general surgical experience prior to our course (e.g., in the context of clinical traineeships, work shadowing, internships, or other student courses at the TU Munich). As other surveys have also shown, the choice of specialization is often based on this stage of studies. 23 24 In a study by Mauch et al, participants stated that early exposure played a key role in strengthening their enthusiasm for microsurgery, specifically. 25 Bechara et al also state that career decisions are based on how long and when different options appeared in the medical training and school rotations. 26 This coincides with our results, which showed that 50.4% stated that the microsurgery course had influenced their future medical direction or career (18.5% stated no influence, 31.1% were unsure), and in general 75.8% were motivated to practice surgery in the future. As our course, especially in the semester-accompanying model, has long-lasting and regularly recurring course contact, this type of influence is plausible and easy to understand.

In this particular course, in addition to the elaborate teaching of surgical skills on selected microsurgical models, the focus is also on teaching the theoretical background. Correct handling of precise instruments and fragile tissue is essential. Since careful microsurgical dissection differs from macroscopic surgery, we do not consider it a disadvantage that some students have no previous surgical knowledge. In the early stages of microsurgical learning, even home practice is beneficial, as described by Malik et al. 7 The basics of needle guidance and performing a single-knot suture are taught using the latex glove or the orange model, both very suitable for these basic skills. 2 27 28 Suturing techniques (end-to-end or end-to-side) are taught on the chicken thigh and the porcine heart coronary. Both models have advantages and disadvantages. The preparation of pig heart coronary arteries can be challenging, whereas the preparation and anastomosis in the chicken model closely resemble the real situation in humans. It was not surprising that 81.7% of participants retrospectively described the chicken model as their favorite. The pig heart was rated as the most difficult by 41.2%, followed by the orange model at 29.4%. In both models, the main focus is on dissection and correct, atraumatic handling of tissue. From a mental perspective, the evaluation is easy to understand as the vessels and nerves in the chicken thigh model are freed from the loose surrounding fat with less stress, making anastomosis or nerve coaptation faster. The sense of achievement is thus safer and faster. In the literature, the chicken model is one of the most popular models and is also described as a pulsatile model, which can provide an even more realistic impression. 29 Although we have not yet established a pulsatile chicken model for teaching the basics, it is part of an advanced course for former participants. Additionally, Fleurette et al showed that students trained on the chicken thigh sometimes performed significantly better in the live rat model than those trained on the rat. 30 Esanu et al came to a similar conclusion and showed that using low and moderate fidelity models was not inferior. 31 This underscores the importance of realistic vessel handling and preparation, which can be transferred from the chicken to the rat model without apparent problems.

A notable finding is that all course participants (100%) felt capable of performing a microsurgical suture after both the 10 to 12 course units of 2 hours each during the semester and the block course with 5 units of 3 hours each, regardless of their initial level of surgical training. Additionally, we consider it a success that 87.5% still feel capable of performing a microsurgical anastomosis. What may also have contributed to this good estimation is that 54.2% stated that they used the skills acquired in the course in activities such as a doctoral thesis or clinical work. Interestingly, only 63.9% felt confident in their ability to perform nerve coaptation. This may be due to a lack of success monitoring (all anastomosed vessels were cut open lengthwise and assessed) and the more abstract, longer healing phase of nerves.

Even though our findings do not correlate the timing of participation in the students' educational journey with their later career choices or their self-perceived practical capabilities, we see a steep learning curve in almost all participants with a similar level of microsurgical skill by the end of each course. To further enhance these skills an advanced course with additional training models has recently been established, subject to follow-up studies in the future. Microsurgical skills can be taught at any point during one's medical study, in some individual cases even at a premedical level, achieving results that are as good or even better than those of experienced residents, while also increasing aspirations toward a surgical career later on. 15 32

### Influence on Future Medical Focus or Career

Two key factors set this study apart from many others analyzing microsurgical training. First, this is a course for medical students only, and we know that the level of surgical training does not significantly affect performance in various microsurgical models. Indeed, in some cases, students with no surgical background performed better at the end of the course than those who had already acquired surgical dexterity. The plasticity of the brain generally favors early learning of these skills, similar to learning in sports. Second, the study analyzes the longevity and specific influence of this course on the further career development of course graduates.

An analysis of the impact of a surgical training course in medical education is rarely found in the literature, although the significance of the findings is substantial. The fact that our course motivated 75.8% of the participants to become surgeons underscores the importance of quality courses. Additionally, such a course can significantly contribute to promoting one's specialty. For example, 60% of participants could imagine doing an internship in oral and maxillofacial surgery, and 37.5% completed their elective traineeship in their practical year. Customized student courses can significantly improve technical skills and positively influence students' career aspirations toward surgery. 33 Such hands-on workshops can play a crucial role in medical education by bridging the gap between theoretical knowledge and practical skills and potentially shaping the future of aspiring physicians. For small surgical specialties in particular, a practical hands-on course of this kind can ensure the next generation of enthusiastic, motivated young individuals who pursue an elective subject in their free time. Furthermore, the practical year, internships, and electives were the top three factors significantly influencing the choice of specialty later on. This underlines the importance of offering such courses and justifies the time spent optimizing the theoretical and practical course content. It is also promising in that future revisions of training and medical licensing regulations will place more emphasis on the practical training of students.

From the authors' perspective, the sustainability of the acquired skills and the significant influence on the students' later careers justify the investment in such an equipment- and personnel-intensive course. It is worth the effort.

## Conclusion

This highly specialized elective course for medical students appears to have a positive influence and long-term impact on their future careers, making the effort worthwhile. Students benefit from early microsurgical education and can transfer the acquired skills to their later professional practice.
